# Jacksonian Epilepsy in Idiopathic Epilepsy

**Published:** 1910-08-13

**Authors:** 


### JACKSONIAN EPILEPSY IN IDIOPATHIC EPILEPSY.

The question of the differential diagnosis of Jacksonian epilepsy, due to tumour or other gross, and possibly operable, lesions of the motor zone, from idiopathic epilepsy, is one of great moment. Some go so far as to say that the commonest cause of Jacksonian spasm is idiopathic epilepsy. In several cases in recent years operations have been done without revealing any tangible lesion, the diagnosis of the nature and site of the lesion having been chiefly made because of the characteristics exhibited by the Jacksonian spasm. In some of these cases a small undiscoverable growth was present in the subcortex, or just outside the limits of the cerebral surface exposed, demonstrated by subsequent developments, by a second operation, or by necropsy. In other cases observation of the patient over a considerable time, as well as a more thorough consideration of all the features of the symptomatology of the case, have made it clear that the Jacksonian spasm, although apparently typical, was in reality simply an integral part of the attack of true idiopathic epilepsy. In rare instances the idiopathic case shows almost restricted Jacksonian manifestations—at least the spasm continues to be largely a hemiepilepsy. It is extremely rare in an idiopathic case for the spasm to remain limited to one limb, or to one side of the face, although this is not unknown, and patients may have abortive attacks of epilepsy with a sensory or sensorimotor manifestation of a very transient character in a part to which the aura of a completed attack is commonly referred. In some cases in which Jacksonian spasm occurs as a part of the manifestation of an idiopathic epileptic attack, the Jacksonian spasm occurs, so to speak, inside the general convulsion; in other words, the patient during the seizure has clonic and tonic spasm which involves more or less regularly all parts of the body, but in which the spasm shows itself most pronouncedly in the limbs or in the face and limb or limbs of one side of the body.

